# Veterans Affairs Medical Center Racial and Ethnic Composition and Initiation of Anticoagulation for Atrial Fibrillation

**DOI:** 10.1001/jamanetworkopen.2024.18114

**Published:** 2024-06-24

**Authors:** Utibe R. Essien, Nadejda Kim, Leslie R. M. Hausmann, Donna L. Washington, Maria K. Mor, Terrence M. A. Litam, Taylor L. Boyer, Walid F. Gellad, Michael J. Fine

**Affiliations:** 1 Veterans Affairs Health Systems Research Center for the Study of Healthcare Innovation, Implementation and Policy, Greater Los Angeles Veterans Affairs Healthcare System, California; 2 Division of General Internal Medicine and Health Services Research, David Geffen School of Medicine at the University of California, Los Angeles; 3 Center for Health Equity Research and Promotion, Veterans Affairs Pittsburgh Healthcare System, Pennsylvania; 4 Division of General Internal Medicine, University of Pittsburgh School of Medicine, Pennsylvania; 5 Department of Biostatistics, University of Pittsburgh Graduate School of Public Health, Pennsylvania

## Abstract

**Importance:**

Racial and ethnic disparities exist in anticoagulation therapy for atrial fibrillation (AF). Whether medical center racial and ethnic composition is associated with these disparities is unclear.

**Objective:**

To determine whether medical center racial and ethnic composition is associated with overall anticoagulation and disparities in anticoagulation for AF.

**Design, Setting, and Participants:**

Retrospective cohort study of Black, White, and Hispanic patients with incident AF from 2018 to 2021 at 140 Veterans Health Administration medical centers (VAMCs). Data were analyzed from March to November 2023.

**Exposure:**

VAMC racial and ethnic composition, defined as the proportion of patients from minoritized racial and ethnic groups treated at a VAMC, categorized into quartiles. VAMCs in quartile 1 (Q1) had the lowest percentage of patients from minoritized groups (ie, the reference group).

**Main Outcomes and Measures:**

The odds of initiating any anticoagulant, direct-acting oral anticoagulant (DOAC), or warfarin therapy within 90 days of an index AF diagnosis, adjusting for sociodemographics, medical comorbidities, and facility factors.

**Results:**

The cohort comprised 89 791 patients with a mean (SD) age of 73.0 (10.1) years; 87 647 (97.6%) were male, 9063 (10.1%) were Black, 3355 (3.7%) were Hispanic, and 77 373 (86.2%) were White. Overall, 64 770 individuals (72.1%) initiated any anticoagulant, 60 362 (67.2%) initiated DOAC therapy, and 4408 (4.9%) initiated warfarin. Compared with White patients, Black and Hispanic patients had lower rates of any anticoagulant and DOAC therapy initiation but higher rates of warfarin initiation across all quartiles of VAMC racial and ethnic composition. Any anticoagulant therapy initiation was lower in Q4 than Q1 (69.8% vs 74.9%; adjusted odds ratio [aOR], 0.80; 95% CI, 0.69-0.92; *P* < .001). DOAC and warfarin initiation were also lower in Q4 than in Q1 (DOAC, 69.4% vs 65.3%; aOR, 0.85; 95% CI, 0.74-0.97; *P* < .001; warfarin, 5.4% vs 4.5%; aOR, 0.82; 95% CI, 0.67-1.00; *P* < .001). In adjusted models, patients in Q4 were significantly less likely to initiate any anticoagulant therapy than those in Q1 (aOR, 0.88; 95% CI, 0.78-0.99). Patients in Q3 (aOR, 0.75; 95% CI, 0.60-0.93) and Q4 (aOR, 0.69; 95% CI, 0.55-0.87) were significantly less likely to initiate warfarin therapy than those in Q1. There was no significant difference in the adjusted odds of initiating DOAC therapy across racial and ethnic composition quartiles. Although significant Black-White and Hispanic-White differences in initiation of any anticoagulant, DOAC, and warfarin therapy were observed, interactions between patient race and ethnicity and VAMC racial composition were not significant.

**Conclusions and Relevance:**

In a national cohort of VA patients with AF, initiation of any anticoagulant and warfarin, but not DOAC therapy, was lower in VAMCs serving more minoritized patients.

## Key Points

**Question:**

Among patients with atrial fibrillation treated in the Veterans Health Administration (VA), is there an association between facility racial and ethnic composition and anticoagulant initiation?

**Findings:**

In this cohort study of 89 791 VA patients with atrial fibrillation from 2018 to 2021, significantly lower receipt of any anticoagulant and warfarin therapy among patients cared for at minority-serving VA medical centers was observed. No significant differences were observed in initiation of direct oral anticoagulant therapy when comparing minority-serving VA medical centers with those serving fewer minority patients.

**Meaning:**

These findings suggest interventions to improve anticoagulant prescribing at minority-serving facilities may improve quality and equity of atrial fibrillation care in the VA.

## Teaser

This cohort study evaluates whether Veterans Health Administration medical center racial and ethnic composition is associated with disparities in anticoagulation for atrial fibrillation.

## Introduction

Atrial fibrillation (AF) is the most common cardiac rhythm disorder in the US, affecting up to 6 million adults.^1,2^ AF is associated with increased cardiovascular mortality and morbidity, including higher risk of ischemic stroke.^3,4^ Oral anticoagulant (OAC) therapy significantly reduces the risk of ischemic stroke in individuals with AF and is the standard of care for patients with moderate to high stroke risk.^5,6^

Prior research has demonstrated access to anticoagulant therapies is inequitable and has been seen as a possible driver for observed racial and ethnic disparities in ischemic stroke among those with AF.^7^ Several studies have described lower prescribing of overall anticoagulation and especially newer, guideline-recommended direct oral anticoagulant (DOAC) therapy in individuals from minoritized racial and ethnic groups, including Black and Hispanic individuals with AF.^8,9^ These findings have also been observed in the Veterans Health Administration (VA), the largest integrated health system, which generally reduces barriers to health care access and provides medications with low or no prescription copayments through a national formulary.^10,11^ Whereas most prior studies have focused primarily on patient and limited clinician-level factors as possible determinants of racial and ethnic prescribing disparities, less is known about how health system-level factors are associated with equitable access to anticoagulation for AF.^12^

Racial segregation, along with the resulting segregation of individuals from minoritized racial and ethnic groups in a small number of health care facilities, may contribute to disparities in health and health care. For example, policies that drive residential segregation and disinvestment in low income areas and neighborhoods where individuals from minoritized groups live also affect access to high-quality health care and the resources available to health care clinicians who serve those areas.^13,14,15^ Prior non-VA studies have shown that health systems that care for higher proportions of individuals from minoritized backgrounds provide lower quality health care, including in cardiovascular disease, and that minoritized patients at these health systems have poorer health outcomes compared with White patients.^16,17,18^ Thus, we sought to determine whether there is an independent association between medical center racial and ethnic composition and initiation of anticoagulant therapy for patients with incident AF. In secondary analyses, we assessed whether there are individual-level racial and ethnic differences in the observed associations between medical center racial and ethnic composition and initiation of anticoagulant therapy, as well as the interaction between these 2 factors. We hypothesized that we would observe lower rates of overall anticoagulant prescribing at medical centers with higher proportions of minoritized patients.

## Methods

We conducted a retrospective cohort study in the previously described Race, Ethnicity and Anticoagulant Choice in Atrial Fibrillation (REACH-AF) cohort.^10,19^ This analysis included patients with incident nonvalvular AF treated at all VA medical centers (VAMCs) nationwide (140 centers) and their associated community-based outpatient clinics from 2018 to 2021. The institutional review board at the VA Pittsburgh Healthcare System approved the study and granted a waiver of informted consent as data in the cohort were deidentified. We followed the Strengthening the Reporting of Observational Studies in Epidemiology (STROBE) reporting guidelines.

### Data Sources

We used administrative and clinical data from the VA Corporate Data Warehouse (CDW) to develop the analytic cohort and create study variables. The CDW contains information on outpatient and inpatient clinical encounters, patient sociodemographics, diagnosis codes, prescribed medications dispensed by VA pharmacies, and VAMC characteristics. We used CDW along with data from the Centers for Medicare and Medicaid Services (CMS) to identify individual race and ethnicity.

### Cohort Eligibility Criteria

**Figure 1.**
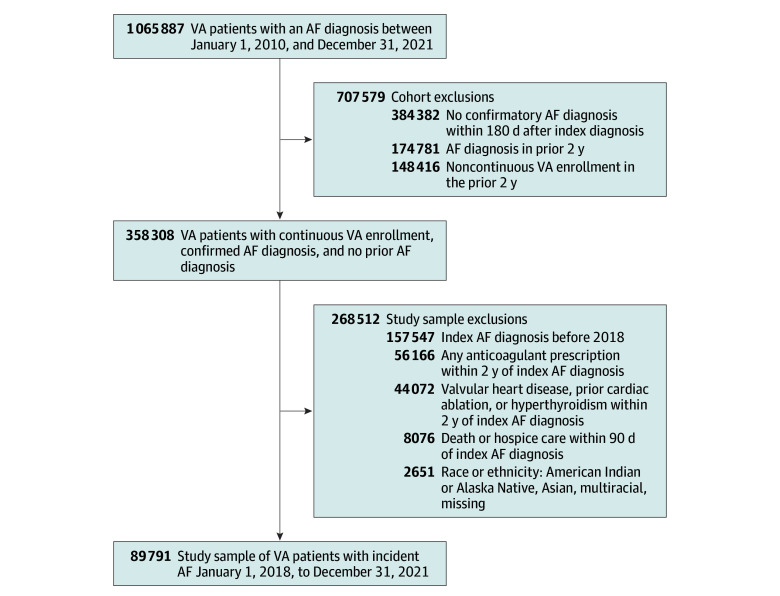
Identification of the Study Sample Among 1 065 887 patients in the VA with an index AF diagnosis from 2010 through 2021, 358 308 patients with continuous VA enrollment, no prior AF diagnosis in the prior 2 years, and a confirmatory AF diagnosis within 180 days after their index diagnosis were identified. After applying additional exclusion criteria, the study cohort included 89 791 patients with incident AF from 2018 to 2021. All exclusions were performed sequentially. AF indicates atrial fibrillation; VA indicates Veterans Health Administration.

We used established *International Statistical Classification of Diseases and Related Health Problems, Tenth Revision* diagnosis codes for AF (ie, I48.0, I48.1, I48.2, and I48.9) from VA outpatient clinical encounters to identify patients with an initial, or index, AF diagnosis between January 1, 2018, and December 31, 2021.^10,20,21^ To define the REACH-AF cohort, we sequentially excluded patients who had any prior outpatient AF diagnosis or were not enrolled in the VA for 2 years before their index diagnosis, and those without 1 or more outpatient confirmatory diagnoses of AF in the VA 7 to 180 days after their index diagnosis (Figure 1). To define the study sample, we excluded patients with valvular AF using diagnosis codes for any form of aortic or mitral valvular disease, repair, or replacement 2 years before the index AF diagnosis.^10^ We excluded patients with diagnoses of cardiac ablation or hyperthyroidism or use of any anticoagulant therapy in the 2 years before AF diagnosis to ensure eligibility for all forms of anticoagulant therapy.^22^ We excluded patients who died or received hospice care 90 days after AF diagnosis. We excluded patients with missing race information (526 patients) or who identified their race as American Indian or Alaska Native, Asian, or multiracial (2125 patients) due to limited sample sizes of these groups at the VAMC level (Figure 1).

### Study Outcomes

We assessed initiation of any form of anticoagulant therapy, defined as the first outpatient prescription for warfarin or DOAC therapy that was filled in the VA within 90 days of the index AF diagnosis. DOAC therapy included a prescription for apixaban, dabigatran, edoxaban, or rivaroxaban.

### Independent Variable and Covariates

VAMC racial and ethnic composition was the independent variable of interest and was defined at the facility level as the proportion of all patients (regardless of AF diagnosis) within a VAMC that identified as non-Hispanic American Indian or Alaska Native, Asian, Black, and Hispanic. We used VA administrative data on self-identified race and ethnicity, supplemented by CMS data when missing, to define the racial and ethnic groups previously listed as well as non-Hispanic White individuals. As noted previously, individuals from American Indian or Alaska Native, Asian, or multiracial identities were excluded given low sample sizes at the VAMC level. VAMC racial and ethnic composition was categorized into quartiles, with VAMCs in quartile 1 (Q1) having the lowest percentage of patients from minoritized racial and ethnic groups (≤10.1%) and serving as the reference group. VAMCs in quartile 4 (Q4) had the highest percentage of patients from minoritized racial and ethnic groups (≥40.4%) and were considered minority-serving VAMCs.

Informed by a health equity research conceptual framework that identifies patient-, clinician-, and facility-level characteristics as potential determinants of disparate care along with prior anticoagulation and AF disparity studies, we assessed baseline patient and facility-level characteristics that are potential confounders of the associations between VAMC racial and ethnic composition and our study outcome.^10,23^ Patient sociodemographic characteristics were age at AF diagnosis, sex, geographic region of the place of residence (ie, Midwest, Northeast, South, West, and US Territories), and area deprivation index (ADI), a measure of neighborhood disadvantage that comprises 17 components of socioeconomic status, including income, education, housing quality, and employment.^24^ Both geographic region and ADI were included in our analysis given prior work demonstrating their association with anticoagulant prescribing.^19,25^ Baseline patient clinical characteristics consisted of select cardiometabolic medical comorbidities (eg, hypertension, diabetes, vascular disease, and congestive heart failure).^10,22^ We quantified 1-year stroke risk in AF using the validated CHA_2_DS_2_VASc score (low, 0-1; moderate, 2-4; and high, >4), which includes congestive heart failure, hypertension, being aged more than 75 years, diabetes, prior stroke, transient ischemic attack, or thromboembolism, vascular disease, being aged 65 to 74 years, and female sex.^26^ We included the variables of the HAS-BLED prediction rule (which includes hypertension, abnormal kidney and/or liver function, stroke, bleeding history, elderly, and concomitant drugs and/or alcohol use) to assess 1-year bleeding risk in AF that were not otherwise included in the previously mentioned variables, including history of kidney or liver disease, bleeding, and use of medications predisposing to bleeding, such as antiplatelets and nonsteroidal anti-inflammatory drugs.^27^ We also recorded the calendar year of all index AF diagnoses. Finally, we included facility variables including the clinical site associated with the index AF diagnosis (eg, cardiology, emergency department, pharmacy, and primary care) as well as a validated variable for facility complexity rating, which was categorized into 3 groups (1, highest complexity; 2, medium; and 3, low) based on a VAMC’s patient volume, number and breadth of physician specialists, patient case mix, intensive care unit (ICU) capabilities, degree of teaching (including number of resident trainee slots) and research services, and administrative complexity.^28,29^ VAMCs with the highest complexity have the highest patient volume, patient risk, highest level of teaching and research, and highest level of ICUs compared with those with the lowest complexity.^30,31^

### Statistical Analysis

We used χ^2^ tests to compare baseline patient and facility characteristics and unadjusted anticoagulant therapy use across quartiles of VAMC racial and ethnic composition, overall, and by individual race and ethnicity. We used mixed logistic regression modeling, with VAMC site as a random effect, to estimate the odds of receipt of (1) any anticoagulant (ie, warfarin and DOAC therapy), (2) DOAC therapy, or (3) warfarin therapy by VAMC racial and ethnic composition quartile. The analysis includes 1 observation for each individual at each site. We adjusted for individual race and ethnicity; demographics; clinical factors, including stroke and bleeding risk; year of AF diagnosis; geographic region; ADI; site of AF diagnosis; and facility complexity. We tested a main effect for individual race and ethnicity, and in secondary analyses, examined the interaction between individual race and ethnicity and quartile of VAMC racial and ethnic composition. We used a 2-tailed *P* value less than .05 to define statistical significance and conducted all analyses using SAS Enterprise Guide version 8.3 (SAS Institute). Data were analyzed from March to November 2023.

## Results

### Baseline Patient Characteristics

Our final cohort comprised 89 791 patients with incident AF at 140 VAMCs from 2018 to 2021; 87 647 patients (97.6%) were male, 9063 (10.1%) were Black, 3355 (3.7%) were Hispanic, 77 373 (86.2%) were White, and the mean (SD) age was 73.0 (10.1) years (Table). Patients in Q4 (ie, VAMCs with the higher proportion of individuals from minoritized groups, or minority-serving) were younger, had higher rates of female patients, and had similar stroke and bleeding risks to those in Q1. Patients in minority-serving VAMCs were significantly more likely to receive their care at high-complexity facilities and more likely to reside in the South (Table).

**Table.** Baseline Characteristics of Patients With Incident Atrial Fibrillation by Quartile of Racial and Ethnic Composition^a^

Sociodemographic characteristics	Patients, No. (%)				
	Quartile 1 (n = 18 141)	Quartile 2 (n = 23 693)	Quartile 3 (n = 28 178)	Quartile 4 (n = 19 779)	Total (N = 89 791)
Age at diagnosis, mean (SD), y	74.0 (9.8)	74.0 (10.0)	73.0 (10.3)	73.0 (10.5)	73.0 (10.2)
≤64	2405 (13.3)	3330 (14.1)	4636 (16.5)	3638 (18.4)	14 009 (15.6)
65-74	7804 (43.0)	10 107 (42.7)	11 991 (42.6)	8235 (41.6)	38 137 (42.5)
75-84	5166 (28.5)	6428 (27.1)	7485 (26.6)	5149 (26.0)	24 228 (27.0)
≥85	2766 (15.2)	3828 (16.2)	4066 (14.4)	2757 (13.9)	13 417 (14.9)
Sex					
Female	369 (2.0)	505 (2.1)	750 (2.7)	520 (2.6)	2144 (2.4)
Male	17 772 (98.0)	23 188 (97.9)	27 428 (97.3)	19 259 (97.4)	87 647 (97.6)
Race and ethnicity					
Black	325 (1.8)	1253 (5.3)	3140 (11.1)	4345 (22.0)	9063 (10.1)
Hispanic	122 (0.7)	306 (1.3)	972 (3.4)	1955 (9.9)	3355 (3.7)
White	17 694 (97.5)	22 134 (93.4)	24 066 (85.4)	13 479 (68.1)	77 373 (86.2)
Region					
Midwest	9910 (54.6)	8612 (36.3)	4067 (14.4)	514 (2.6)	23 103 (25.7)
Northeast	2885 (15.9)	7316 (30.9)	1877 (6.7)	1418 (7.2)	13 496 (15.0)
South	3411 (18.8)	4131 (17.4)	15 131 (53.7)	13 137 (66.4)	35 810 (39.9)
West	1705 (9.4)	3396 (14.3)	6900 (24.5)	3839 (19.4)	15 840 (17.6)
US Territories	NA	NA	5 (<.01)	494 (2.5)	499 (0.6)
Area deprivation index, %^b^					
Quintile 1 (1-29)	1356 (7.5)	3462 (14.6)	5943 (21.1)	4853 (24.5)	15 614 (17.4)
Quintile 2 (30-45)	2915 (16.1)	4540 (19.2)	5484 (19.5)	3284 (16.6)	16 223 (18.1)
Quintile 3 (46-61)	3953 (21.8)	5194 (21.9)	5471 (19.4)	3406 (17.2)	18 024 (20.1)
Quintile 4 (62-77)	4577 (25.2)	4910 (20.7)	5012 (17.8)	3348 (16.9)	17 847 (19.9)
Quintile 5 (78-100)	5237 (28.9)	5434 (22.9)	6137 (21.8)	4724 (23.9)	21 532 (24.0)
Clinical site of atrial fibrillation diagnosis					
Primary care	9242 (50.9)	11 723 (49.5)	14 200 (50.4)	9184 (46.4)	44 349 (49.4)
Cardiology	1673 (9.2)	2582 (10.9)	3689 (13.1)	2808 (14.2)	10 752 (12.0)
Emergency department	2358 (13.0)	2860 (12.1)	3423 (12.1)	2595 (13.1)	11 236 (12.5)
Pharmacy	3368 (18.6)	4237 (17.9)	4314 (15.3)	3362 (17.0)	15 281 (17.0)
Other	1500 (8.3)	2291 (9.7)	2552 (9.1)	1830 (9.3)	8173 (9.1)
Year of atrial fibrillation diagnosis					
2018	5183 (28.6)	6833 (28.8)	8107 (28.8)	6122 (31.0)	26 245 (29.2)
2019	5016 (27.7)	6720 (28.4)	8089 (28.7)	5796 (29.3)	25 621 (28.5)
2020	3516 (19.4)	4721 (19.9)	5374 (19.1)	3516 (17.8)	17 127 (19.1)
2021	4426 (24.4)	5419 (22.9)	6608 (23.5)	4345 (22.0)	20 798 (23.2)
Clinical characteristics					
Selected medical comorbidities					
Congestive heart failure	2693 (14.8)	3618 (15.3)	4513 (16.0)	3395 (17.2)	14 219 (15.8)
Hypertension	13 528 (74.6)	17 462 (73.7)	20 969 (74.4)	14 782 (74.7)	66 741 (74.3)
Diabetes	6630 (36.5)	8678 (36.6)	10 536 (37.4)	7692 (38.9)	33 536 (37.3)
Vascular disease	6767 (37.3)	9042 (38.2)	10 283 (36.5)	7080 (35.8)	33 172 (36.9)
Prior bleeding	2156 (11.9)	3136 (13.2)	3810 (13.5)	2620 (13.2)	11 722 (13.1)
Liver disease	936 (5.2)	1348 (5.7)	1753 (6.2)	1275 (6.4)	5312 (5.9)
Kidney disease	1373 (7.6)	1938 (8.2)	2615 (9.3)	1811 (9.2)	7737 (8.6)
Medications predisposed to bleeding	8630 (47.6)	11 178 (47.2)	14 148 (50.2)	10904 (55.1)	44860 (50.0)
CHA_2_DS_2_-VASc Stroke Risk Score^c^					
Low (0-1)	2519 (13.9)	3339 (14.1)	4174 (14.8)	3041 (15.4)	13 073 (14.6)
Moderate (2-4)	12 062 (66.5)	15 582 (65.8)	18 344 (65.1)	12 616 (63.8)	58 604 (65.3)
High (>4)	3560 (19.6)	4772 (20.1)	5660 (20.1)	4122 (20.8)	18 114 (20.2)
Facility characteristics					
Facility complexity level					
High (1)	9316 (51.4)	17 686 (74.6)	25 440 (90.3)	18 466 (93.4)	70 908 (79.0)
Medium (2)	4052 (22.3)	3450 (14.6)	1612 (5.7)	994 (5.0)	10 108 (11.3)
Low (3)	4773 (26.3)	2557 (10.8)	923 (3.3)	316 (1.6)	8569 (9.5)

Abbreviation: NA, not applicable.

^a^ Veterans Health Administration medical center (VAMC) racial and ethnic composition was categorized into quartiles, with VAMCs in quartile 1 having the lowest percentage of individuals from minoritized racial and ethnic groups (≤10.1%) and VAMCs in quartile 4 having the highest percentage of individuals from minoritized racial and ethnic groups (≥40.4%) and were considered minority-serving VAMCs.

^b^ Area deprivation index scores are categorized into quintiles using percentile rankings of all US neighborhoods from least (first percentile) to most (one hundredth percentile) disadvantaged.

^c^ CHA_2_DS_2_-VASc indicates a score composed of points for congestive heart failure; hypertension; age 75 years; diabetes; prior stroke, transient ischemic attack, or thromboembolism; vascular disease; age 65 to 74 years; and sex category (female).

### Initiation of Any Anticoagulant Therapy by VA Medical Center Racial and Ethnic Composition

**Figure 2.**
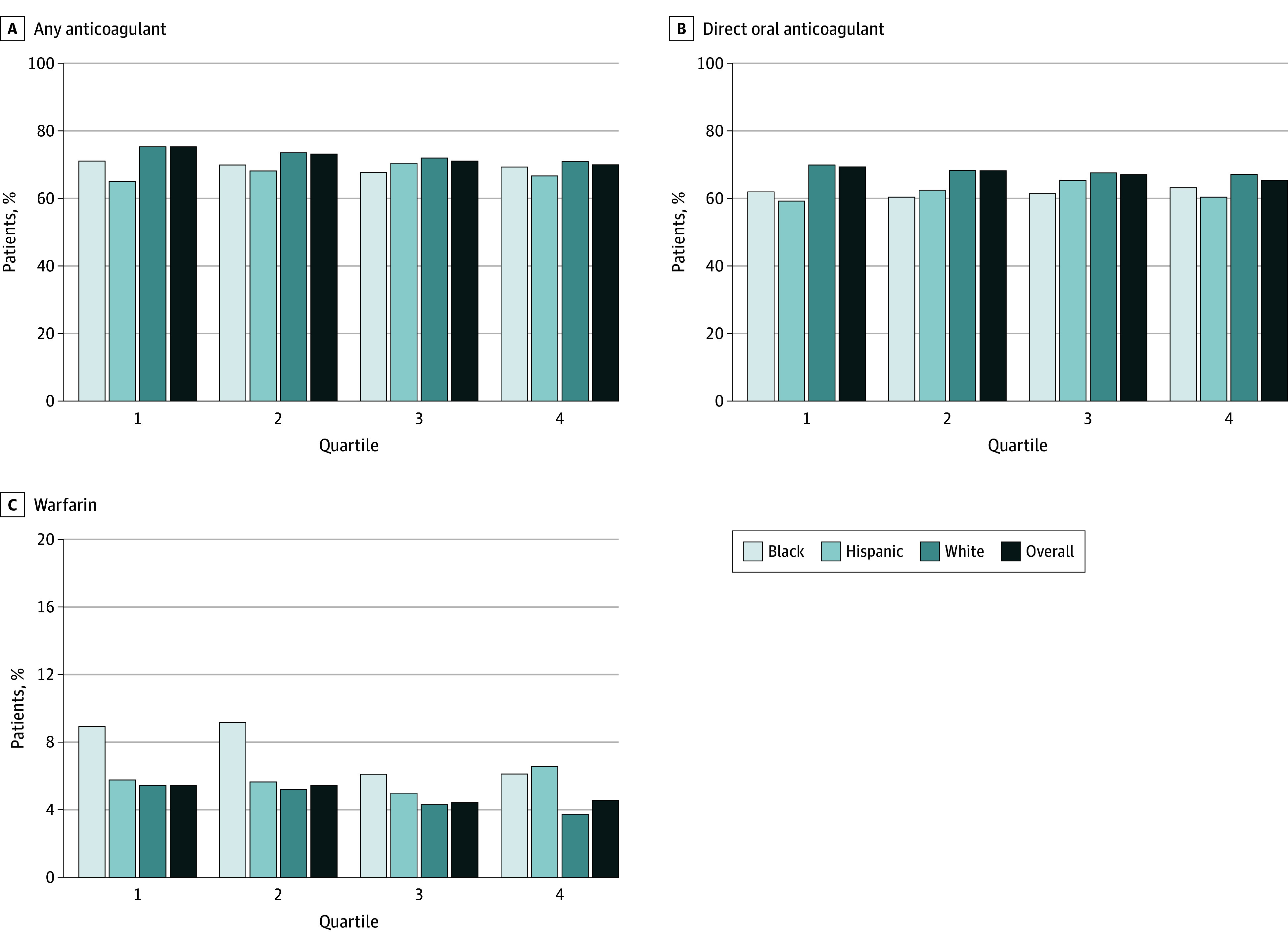
Frequency of Anticoagulation for Patients With Incident Atrial Fibrillation by Quartile of Veterans Health Administration (VA) Medical Center Racial and Ethnic Composition, Overall and by Race and Ethnicity A, The unadjusted rates of any oral anticoagulant therapy; B, direct oral anticoagulant therapy; and C, warfarin therapy initiation are displayed across quartiles (Q) of VA medical center racial and ethnic composition, overall and by race and ethnicity (Black, Hispanic, and White). Q1 represents VA medical centers with the lowest proportion of patients from minoritized racial and ethnic groups, and Q4 represents VA medical centers with the highest proportion of minoritized patients.

**Figure 3.**
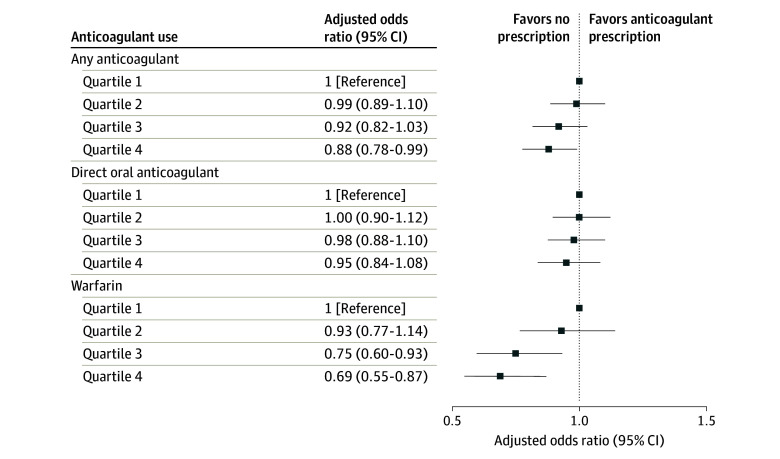
Association of Veterans Health Administration (VA) Medical Center Racial and Ethnic Composition and Anticoagulation for Patients With Incident Atrial Fibrillation In logistic regression modeling, adjusted by patient clinical and socioeconomic, clinician, and facility-level factors, VA medical centers in quartile 4 (ie, minority-serving) had significantly lower initiation of any anticoagulant compared with those in quartile 1. VA medical centers in quartiles 3 and 4 had significantly lower initiation of warfarin compared with those in quartile 1. There was no statistically significant difference in direct acting oral anticoagulants initiation across quartiles of racial and ethnic composition.

Overall, 64 770 individuals (72.1%) initiated any anticoagulant therapy across all 4 racial and ethnic composition quartiles. Any anticoagulant therapy was 5.4 percentage points lower for individuals treated in Q4 VAMCs than those treated in Q1 VAMCs (69.8 vs 74.9%; adjusted odds ratio [aOR], 0.80; 95% CI, 0.69-0.92; *P* < .001) (Figure 2A). Compared with individuals treated in Q1 VAMCs, the adjusted odds of initiating any anticoagulant therapy were lower among individuals treated in Q4 VAMCs (aOR, 0.88; 95% CI, 0.78-0.99; *P* < .001) (Figure 3). The unadjusted frequency of any anticoagulant initiation ranged from 1.6 to 4.2 percentage points lower for Black than White patients and from 1.3 to 6.0 percentage points lower for Hispanic than White patients across all quartiles (Figure 2A). Whereas the main effects of the association between Black race (aOR, 0.82; 95% CI, 0.78-0.87) and Hispanic ethnicity (aOR, 0.91; 95% CI, 0.83-0.99) and any anticoagulant initiation were statistically significant, there was no significant race and ethnicity by medical center racial and ethnic composition interaction in our model (χ^2^_6_ = 1.46; *P* = .19), indicating similar Black-White and Hispanic-White differences in prescribing across all 4 quartiles.

### Initiation of DOAC Therapy by VA Medical Center Racial and Ethnic Composition

Overall, 60 362 (67.2%) individuals initiated DOAC therapy among all patients with AF, ranging from 65.3% in Q4 to 69.4% in Q1 (aOR, 0.85; 95% CI, 0.74-0.97; *P* = .02) (Figure 2B). In adjusted models, there was no significant difference in DOAC initiation between patients treated at Q1 VAMCs and Q2 (aOR, 1.00; 95% CI, 0.90-1.12), Q3 (aOR, 0.98; 95% CI, 0.87-1.10), or Q4 (aOR, 0.95; 95% CI, 0.84-1.08) VAMCs (Figure 3). The unadjusted frequency of DOAC initiation ranged from 4.0 to 7.9 percentage points lower for Black than White patients and 2.9 to 10.7 percentage points lower for Hispanic than White patients, respectively, across all quartiles (Figure 2B). The main effects of Black race (aOR, 0.76; 95% CI, 0.71-0.80) and Hispanic ethnicity (aOR, 0.87; 95% CI, 0.81-0.96) on DOAC initiation were statistically significant, yet there was no significant interaction between race and ethnicity and VAMC racial and ethnic composition (χ^2^_6_ = 1.83; *P* = .09).

### Initiation of Warfarin Therapy by VA Medical Center Racial and Ethnic Composition

Overall, 4408 (4.9%) individuals initiated warfarin therapy among all patients with AF. Warfarin initiation ranged from 4.5% to 5.4% between Q1 and Q4 (aOR, 0.82; 95% CI, 0.67-1.00; *P* = .05) (Figure 2C). In adjusted models, patients at Q3 (aOR, 0.75; 95% CI, 0.60-0.93) and Q4 (aOR, 0.70; 95% CI, 0.55-0.87) VAMCs were significantly less likely to initiate warfarin than those in Q1 VAMCs (Figure 3). Unlike DOAC prescribing, the frequency of warfarin initiation was up to 3.9 percentage points higher for Black than White patients and up to 2.8 percentage points higher for Hispanic than White patients (Figure 2C) across quartiles. The main effects of Black race (aOR, 1.53; 95% CI, 1.39-1.70) and warfarin initiation were statistically significant, yet there was no significant interaction between patient race and ethnicity and VAMC racial and ethnic composition (χ^2^_6_ = 0.51; *P* = .74). There was no significant association between Hispanic ethnicity and warfarin initiation.

## Discussion

In a nationwide cohort of individuals with incident AF receiving care at the VA, we observed significantly lower odds of any anticoagulant and warfarin therapy initiation among individuals treated at VAMCs serving larger proportions of minoritized patients. There was no association between initiation of newer DOAC therapy and racial and ethnic composition of VAMCs. We also observed significantly lower rates of any anticoagulant and DOAC therapy initiation for Black and Hispanic than White patients within each of the 4 quartiles of VAMC racial and ethnic composition, while observing higher rates of traditional warfarin therapy initiation for Black patients—racial differences that were similar across all racial and ethnic composition quartiles.

Using contemporary clinical data from the largest safety-net health system in the US, our study is one of the first examinations of the role of health system racial and ethnic composition in the management of AF. Prior large registries,^8,32^ insurance claims databases,^9,33^ and health system–based studies^34^ have reported racial and ethnic differences in receipt of anticoagulant therapy for AF; however, the majority of these studies have examined patient-level sociodemographic and clinical characteristics with little discussion of how site of care is associated with these disparities. A study of hospitalized patients with AF in the Get With the Guidelines—Atrial Fibrillation Registry^7^ found that Black patients with AF were significantly less likely than White patients to be discharged from the hospital with any anticoagulant or DOAC therapy. Although the analysis controlled for health system-level factors such as size, region, rurality, teaching status, and presence of an electrophysiology specialist, racial and ethnic composition of the hospital was not included in the analysis. Similarly, previous work showing lower initiation of any anticoagulant and DOAC therapy among Black and Hispanic individuals with AF compared with White individuals in the VA examined facility factors such as the type of VA medical center, but did not examine racial and ethnic composition as a facility characteristic.^10^ Thus, this analysis extends prior work by examining the potential role of facility racial and ethnic composition on AF management and suggests addressing anticoagulant prescribing practices and increasing availability of resources, including at anticoagulation clinics at minority-serving VAMCs, may serve as intervention targets for eliminating disparities in overall AF care. Furthermore these findings, guided by a multilevel health equity conceptual framework, suggest that interventions to address racial and ethnic differences within a VAMC may differ from those that address disparities between VAMCs with varying facility characteristics, such as racial and ethnic composition.

Beyond AF, prior research has examined the role of health system minority composition on health outcomes, demonstrating higher rates of cardiovascular mortality,^35^ worse outcomes after hospitalization for cardiac arrest,^36^ and poorer access to palliative care at end of life^37^ in health systems with larger populations of minoritized patients. However, few studies have specifically explored how this factor may serve as a determinant of medication prescribing disparities. An analysis of Medicare beneficiaries hospitalized for an acute myocardial infarction from 1995 to 2014 found that Black patients were more likely to use hospitals with poorer survival rates compared with White patients.^38^ Furthermore, the study found that Black patients were more likely to receive care at health systems with lower rates of recommended myocardial infarction treatment (ie, beta blockers and aspirin) compared with White patients. These medication disparity findings are similar to those observed in our analysis and suggest that inequitable implementation of guideline-based therapies at the health system level may play an important role in persistent cardiovascular outcome disparities. While most prior analyses have focused on hospital minority composition, previous work has also reported an association between outpatient clinics serving a higher proportion of patients from minoritized racial and ethnic groups and poorer health outcomes, including demonstrating higher rates of depression, substance use disorders, and chronic pain in these clinical settings.^39^

The reasons for the differences in care observed across strata of VAMC racial and ethnic composition is likely multifold. Previous research has shown that minoritized patients are more likely to receive their care at lower quality health systems, which may lead to poorer access to high-quality medications.^18^ This lower quality could be the result of decreased access to resources at minority-serving health systems, including limited availability of primary care clinicians, and in the case of AF, anticoagulation pharmacists, or specialist clinicians such as electrophysiologists, as well as differing levels of expertise and experiences by these clinicians in managing AF. Furthermore, availability of services, clinical staffing, and access to high-quality health information technology needed to address anticoagulation needs may be limited at minority-serving health systems.^40^ Beyond clinical determinants of care, differences in the geographic location of care between racial and ethnic groups in the US are likely the result of centuries of structural racism, which resulted in neighborhood segregation through policies such as redlining, restrictive covenants, and discriminatory housing law, which have determined where individuals from minoritized groups reside in the US.^41,42^ Similarly, federal and state-sponsored hospital segregation directed minoritized patients to receive their health care in hospitals separate from White individuals. The legacy of such policies, which were also present in the VA,^43^ have likely had important impacts on equitable access to care between racial and ethnic groups.

Nevertheless, the lack of disparities in access to newer DOAC therapies across strata of racial and ethnic composition observed in our analysis suggests segregation of patients in facilities with overall low DOAC use alone is not driving national disparities in high-quality anticoagulant prescribing for patients with AF in the VA.^10^ This finding represents an urgent need to more directly examine other possible individual patient and clinician or structural determinants of these disparities. The higher rates of warfarin therapy among Black and Hispanic patients across all quartiles also suggest differential rates of deimplementation of an older therapy for stroke prevention. Strategies to improve equitable deprescribing in the VA warrants further study.

### Limitations

There are limitations to our study. First, this is an analysis of patients receiving care in the VA health system and may not be generalizable to non-VA populations. Nonetheless, examining this analysis in the VA is important as it removes much of the financial concerns traditionally present when evaluating medication prescribing disparities. Second, while employing a robust statistical model that included available patient clinical and sociodemographic factors along with facility level factors, there may be unmeasured confounding along with limited capture of social determinants such as income, health literacy, and experiences with health care discrimination that may influence anticoagulant decision-making. Third, due to lower numbers of some racial and ethnic groups in the VA, the analysis was limited to White vs Black and White vs Hispanic differences in anticoagulant prescribing. Prior work has demonstrated national-level disparities in anticoagulation among American Indian or Alaska Native and Asian individuals and future work exploring facility-level differences among these populations is needed.^10,34,44^ Fourth, our analysis was limited to patients receiving prescriptions within the VA without assessment of medications received outside the VA from commercial, Medicare, or Medicaid insurers, although our prior work suggests that such anticoagulant receipt is minimal.^22^ Additionally, our analysis was focused on prescriptions filled and did not assess primary or subsequent medication adherence outcomes.

## Conclusions

In a large national cohort of patients with AF treated in the VA health care system, we found significant differences in any anticoagulant and warfarin therapy at VAMCs that care for a higher proportion of patients from minoritized racial and ethnic groups compared with those with lower proportions. These findings suggest the need to direct effective interventions at VAMCs that serve higher proportions of minoritized patients with AF to increase the prescribing of guideline-recommended anticoagulation and improve overall equity and quality of AF care in the VA.

## Notes

Supplement.
Data Sharing Statement
