# Supplementary material for: Veterans Affairs Medical Center Racial and Ethnic Composition and Initiation of Anticoagulation for Atrial Fibrillation
Source: JAMA Netw Open. 2024 Jun 24;7(6):e2418114. doi: 10.1001/jamanetworkopen.2024.18114 (PMC11197447; doi:10.1001/jamanetworkopen.2024.18114)
Supplement: Supplement. — Data Sharing Statement [file jamanetwopen-e2418114-s001.pdf]

Data Sharing Statement

Essien. Veterans Affairs Medical Center Racial and Ethnic Composition and Initiation of Anticoagulation for Atrial Fibrillation. *JAMA Netw Open*. Published June 24, 2024. doi:10.1001/jamanetworkopen.2024.18114

Data
Data available: No
